# Donkey serum albumin improves cyclophosphamide-induced anemia in mice

**DOI:** 10.1515/biol-2025-1262

**Published:** 2026-04-29

**Authors:** Nan Qin, Yuwang Cao, Ruiqi Xu, Xinyuan Liu, Guofang Zheng, Lulu Song, Rui Zhang, Yunfei Li, Lixia Xing

**Affiliations:** College of Chinese Medicine and Food Engineering, Shanxi University of Chinese Medicine, 030619, Jinzhong, China

**Keywords:** anemia, donkey serum albumin, bone marrow, histopathology

## Abstract

Cyclophosphamide (CP) is commonly used in cancer chemotherapy and the treatment of autoimmune diseases. In clinical applications, it exhibits the side effect of progressive anemia. Donkey serum albumin (DSA) is the most abundant protein component in Asini Corii Colla (ACC). This experiment aims to investigate the alleviating effect of donkey serum albumin on CP-induced anemia. 60 mice were randomly divided into six groups: normal control group (NC), model control group (MC), positive control group (PC), low-dose DSA (DSA-L, 75 mg/kg/d), medium-dose DSA (DSA-M, 150 mg/kg/d), and high-dose DSA (DSA-H, 300 mg/kg/d), with 10 mice in each group. An anemia model was established by intraperitoneal injection of CP. The treatment continued for 21 days. Afterward, the mice were euthanized, and histopathological examinations of bone marrow and spleen tissues were conducted. Organ indices, the number of bone marrow nucleated cells, and the levels of Erythropoietin (EPO), Thrombopoietin (TPO), and Vascular Endothelial Growth Factor (VEGF) in mouse serum and bone marrow supernatant were measured using ELISA. The model group exhibited significantly lower thymus index, White Blood Cell count (WBC), Red Blood Cell count (RBC),Hemoglobin (HGB), Platelet count (PLT), and bone marrow nucleated cell count (*P < 0.01*), as well as significantly reduced levels of EPO, TPO, and VEGF in serum and bone marrow supernatant (*P < 0.01*). Additionally, obvious tissue damage was observed in the bone marrow and spleen of the model group. In contrast, all DSA dose groups showed significant improvements in these indices compared to the model group (*P < 0.05*, *P < 0.01*), along with notable amelioration of histopathological damage in the bone marrow and spleen. Donkey serum albumin significantly improves hematological function in mice with CP-induced anemia.

## 1 Introduction

Anemia is one of the most common clinical situations encountered in the clinical setting. This disease affects people all over the world it is found in more than a quarter of the world’s population [1]. Anemia in Chinese residents has an average prevalence of 20.1 % [2]. Patients commonly present with dizziness, fatigue, drowsiness, and pallor [3], [4], [5]. Cyclophosphamide (CP), a nitrogen mustard alkylating agent, is used in the treatment of cancer chemotherapy and auto-immune disorder. In clinical application, CP exhibits many side effects, including bone marrow suppression and cytotoxicity leading to progressive anemia, oxidative stress and immunological suppression [6].

The treatment methods for anemia have mainly been immuno-suppression and hematopoietic stimulating factors, which come with their own side effects such as [7], 8]. Asini Corii Colla (ACC), a traditional Chinese health product and herbal medicine, is widely used for the treatment of anemia and exhibits minimal toxicity and side effects. Proteins and peptides constitute its primary active components [9]. According to the research, Asini Corii Colla compounded slurry can significantly improve periphery blood in mice, raise the number of bone marrow mononuclear cells, and reverse atrophy in the thymus and spleen with a clear dose-relation seen [10]. Technical progress prompts ACC to change from being in traditional compound formulas and just swallowed as is, into a trend of isolating and using a lot of ACC’s active parts, mainly the active proteins. Donkey serum albumin (DSA), as the most abundant protein in ACC, serves as a key contributor to its hematopoietic function [11]. Li Hao et al. [12], 13]. Observed a clear stimulation of the proliferation of human BMMNCs and mouse BMSCs as well as improvement of hematopoiesis and resistance to chemotherapeutic drugs in mice by peptides derived from DSA.

**Figure 1:**
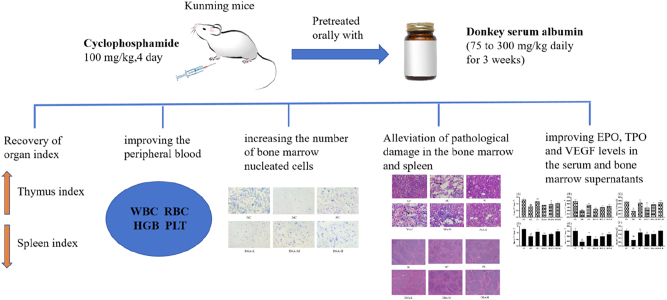
Summary of the experimental design and findings.

We created a mouse with CP-CA anemia to examine if DSA had any potential to treat CA anemia. For each individual group of mice, we took peripheral blood parameters, and analyzed Epo, Tpo, VEGF in bone marrow supernatant and serum. Quantified the bone marrow nucleated cells and studied the bone marrow and spleen tissues, in order to study the role of DSA on treating anemia and looking for new disease prevention techniques (Figure 1).

## 2 Materials and methods

### 2.1 Extraction of DSA

Two grams of donkey hide homogenized powder (80-mesh sieve) were added to 16 ml of a trichloroacetic acid-acetone (1:10) composite solution for dissolution. The precipitate was collected by centrifugation. After drying and grinding, 6 ml of urea lysis buffer was added. The mixture was ultrasonicated for 30 min (25 °C, 480 W), and the suspension was filtered through a membrane to obtain the protein solution. A half-saturated ammonium sulfate solution was added dropwise to salt out the proteins, followed by centrifugation for 15 min (3,000 r/min, 4 °C). The precipitate was discarded, and the supernatant was collected. The crude DSA product was obtained through dialysis and freeze-drying. The DSA solution was further purified using Sephadex G-75 preparative chromatography. Fractions corresponding to the absorption peaks were sampled and collected. Finally, DSA was obtained after vacuum freeze-drying [14], 15].

### 2.2 Establishment and administration of the anemia mouse model

Beijing Huafukang Biotechnology Co., Ltd provided 60 KM female mice, six-week-old, SPF grade, healthy, weighing between 18 and 22 g. The certificate number is as follows: SCXK (Beijing) 2019-008. The mice were given an adaptive diet for 7 days and then were divided randomly into six different groups: The groups were divided randomly as normal control (NC), model control (MC), positive control (PC), low-dose DSA (DSA-L), middle-dose DSA (DSA-M) and high-dose DSA (DSA-H). Each group had 10 animals. The control group was injected with the same volume of normal saline through the intraperitoneal method, and the other groups were given four consecutive daily intraperitoneal injections of 100 mg/kg CP to establish the mouse anemia model [16]. Drug administration was started on the 5th day. Positive control group was given Shengxuening tablets through gavage [17], 18]. The dosage was 400 mg/kg. In group DSA, 75 mg/kg, 150 mg/kg and 300 mg/kg were given by oral, and the equivalent human dose is estimated by the body surface area calculation. Both the normal saline group and the model saline group received the same amount of normal saline. After continuous treatment for 21 days, the mice were killed with cervical dislocation, after which the parameters were measured.

**Ethical approval:** The research related to animal use has been complied with all the relevant national regulations and institutional policies for the care and use of animals, and has been approved by the Experimental Animal Ethics Committee of Shanxi University of Chinese Medicine (Approval No. AWE202508357).

### 2.3 Determination of organ indices in the anemic mice

We took out the spleens and thymuses from the mice after disassembling them, removed them completely, and put them in an ice-cold normal saline solution. Filter water rinse 3 times, dry with new filter paper, and remove normal saline. The spleen and thymus were weighed, and the organ index was calculated by the following formula: organ index = organ mass (mg)/animal mass (g).

### 2.4 Detection of peripheral blood in the anemic mice

Draw blood from subconjunctival retrobulbar venous plexus with heparin sodium as anticoagulant after checking. Sample’s White Blood Cell count (WBC), Red Blood Cell count (RBC), Hemoglobin (HGB), Platelet count (PLT) were all done through an automatic biochemical analyzer.

### 2.5 Detection of the number of bone marrow nucleated cells in the anemic mice

The left femur of each mouse was taken out and the muscle was removed from either end, the ends of them were removed. The bone marrow cavity is repeatedly flushed with RPMI – 1,640 solution to obtain cells, which are subsequently centrifuged at 1,000 r/min for 5 min at 4 degrees Celsius and the supernatant is discarded. It was repeated three times in all. Next take 20 µl of the cells: Microscope was used for the observations and counts of nucleated cells after dropping suspension on blood cell counting plates.

### 2.6 Detection of bone marrow in the anemic mice via Swiss‒Giemsa staining

The left femur of the mouse was cut off at both ends, then bone marrow cell flushing was carried out into bone marrow smears by special fetal bovine serum. The bone marrow damage score was assessed following staining with Wright’s staining solution. Scoring criteria is shown in Table 1.

**Table 1:** Bone marrow injury scoring criteria.

Score	Standard
0	No damage: no lipid droplets, rich in nucleated cells and megakaryocytes.
1	Mild injury: a few lipid droplets, nucleated cells and slightly decreased megakaryocytes.
2	Moderate damage: increased number of lipid droplets and enlarged diameter; decreased number of nucleated cells and megakaryocytes.
3	Severe injury; many lipid droplets and a significant decrease in nucleated cells and megakaryocytes.

### 2.7 Histopathology

BLOOD was taken from the animals on the day prior to euthanizea, and their SPLEENS and FEMURS were removed for use in the later BONE MARROW analysis. collect tissue samples, prepare into hematoxylin and eosin-stained slides, and look at microscope to see if there are any structural changes. Bone marrow injury was assessed according to the criteria in Table 1.

### 2.8 Levels of EPO, TPO and VEGF detected in the serum of the anemic mice via ELISA

Get the ocular blood samples from the animals. Next, take out the sample and spin it at 4 °C for 10 min at 3,500 r/min. the pipettor took and collected the upper serum, separated it, and put it into −80 °C. Serum Erythropoietin (EPO), Thrombopoietin (TPO), and Vascular Endothelial Growth Factor (VEGF) of the mouse were detected using an ELISA kit, according to the kit instructions for higher accuracy.

### 2.9 Levels of EPO, TPO and VEGF detected in the bone marrow supernatants of the anemic mice via ELISA

Breaking mouse femur’s 2 ends and flushed out with PBS. After which it was centrifuged at 4 °C and 2,000 rpm for 10 min. Next, remove the supernatant. We used ELISA kits as per instructions to detect EPO, TPO, and VEGF level in mouse bone marrow.

### 2.10 Statistical analysis

Statistical analysis of SPSS 26.0 was conducted with results stated as -SEM and one-way ANOVA tested the mean difference between the two groups. Statistical significance was set at *P < 0.05*, with *P < 0.01* considered highly significant. GraphPad Prism 6.0 software is used for the visual representations created.

## 3 Results and discussion

### 3.1 Effect of DSA on the organ indices of the anemic mice

**Figure 2:**
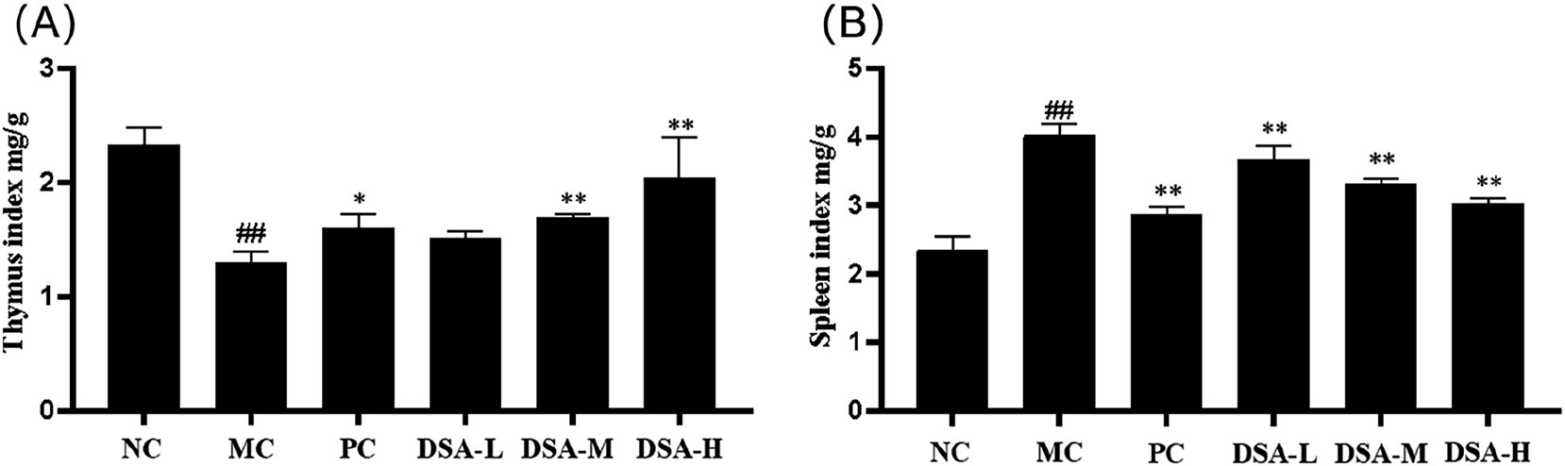
Effect of DSA on the organ indices of the anemic mice. (A) Thymus index; (B) spleen index values are mean ± SD (*n* = 10). Compared with the normal control group: ^##^ *P* < 0.01; compared with the model control group: **P < 0.01, *P < 0.05.

And then the thymus and the spleen are part of our immune systems in our body. Also, serious ischemia, certain pathologies, and spleen and thymus participation in hematopoiesis can be with the help of other organs [19]. From Figure 2 we can see that the spleen index for model control group is much higher than that of normal control group *P* < 0.01, the thymus index is much lower *P* < 0.01, implying that after CP injection, thymus atrophy and declined immune function appeared in mice. Immunosuppression reduces the number of blood cells, causing the spleen to grow and swell. The spleen index in each treatment group was significantly lower than the model control group (*P* < 0.01), but the thymus index of the positive control group and the medium- and high-dose DSA groups were significantly higher than those of the model control group (*P* < 0.01, *P* < 0.05). These results indicate that DSA ameliorates CP-induced splenic hypertrophy and thymic atrophy, thereby supporting hematopoietic recovery.

### 3.2 Effect of DSA on the peripheral hemogram of the anemic mice

Peripheral blood cell counts make the human body’s blood. Peripheral blood shows whole blood cell count is low, white blood cell count is also low. RBC count helps see if someone making too many or too much red cells. It would be a signal that HGB counts lower than normal would give your body too little red pigment that makes your blood, it’s unable to do a solid job moving oxygen through and maybe even make the whole thing run low on resources needed to function regularly. Table 2 showed that the WBC, RBC, HGB, PLT in Model control were much less than the normal control group (*P* < 0.01) There are certain alterations in the mouse’s peripheral blood; blood cells counts are very low. Successfully created mouse anemia. In the model control group, the WBC and RBC were less than the treatment group respectively, and both differences were statistically significant (*P* < 0.001, *P* < 0.01). The hgb level of medium and high-dose DSA group have obvious rise than the pos group, the hgb rise up 1.39, 1.76 times respectively compared to the pos group (*P* < 0.01). PLT positive control group and high dose DSA group is significantly increased, with *P* < 0.01 and *P* < 0.05 respectively. It means that DSA is improving the peripheral blood in mice with anemia.

**Table 2:** Effect of DSA on the peripheral blood in the anemic mice.

Group	Dose mg/(kg·d)	WBC/(×10^9^/L)	RBC/(×10^12^/L)	HGB (g/L)	PLT (×10^9^/L)
NC	–	6.28 ± 1.04	9.96 ± 0.86	204.00 ± 11.36	866.00 ± 119.25
MC	–	1.32 ± 0.43^##^	4.72 ± 0.93^##^	127.00 ± 21.66^##^	306.00 ± 20.42^##^
PC	250	4.68 ± 0.04^**^	9.89 ± 0.74^**^	172.33 ± 5.03^**^	473.67 ± 36.08^**^
DSA-L	75	2.35 ± 0.39^*^	6.59 ± 0.85^*^	145.33 ± 2.31	348.33 ± 17.62
DSA-M	150	3.11 ± 0.14^**^	9.01 ± 0.99^**^	154.67 ± 4.04^**^	396.00 ± 7.94
DSA-H	300	4.32 ± 0.50^**^	9.25 ± 0.12^**^	167.34 ± 2.52^**^	421.67 ± 17.79^*^

Mean ± SD (*n* = 10) was compared with the normal control group: ^#*#*^ *P* < 0.01; and the model control group: **P < 0.01, *P < 0.05.

### 3.3 Effect of DSA on the number of bone marrow nucleated cells in the anemic mice

**Figure 3:**
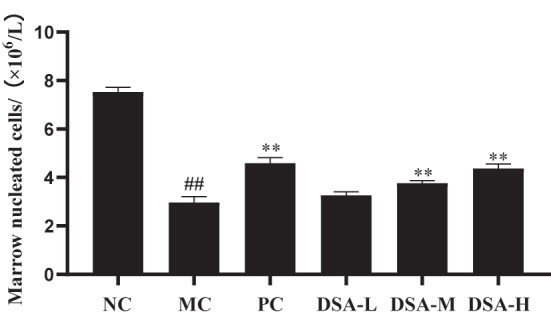
Effect of DSA on the number of nucleated cells in the bone marrow of the anemic mice. Each value is the mean + SD (*n* = 10) compared to the normal control group: ^##^ *P* < 0.01; compared with the model control group: **P < 0.01, **P < 0.05.

The amount of bone marrow that is used to make the blood is based on the number of the no – nucle- ated cells. Bone marrow with fewer nucleated cells: Bone marrow hematopoiesis is disturbed [20]. Figure 3 shows that there is a significant difference in the number of bone marrow nucleated cells between the model control group and the normal control group (*P* < 0.01). This indicates the establishment of an anemia mouse model with bone marrow damage. From the research it was found out that DSA injection into anemic mice increased nucleated cells. Positive control group and medium-high dose DSA groups have obviously higher eukaryotic cell count in bone marrow compare to the model control group. *P* < 0.01 respectively. Low Dose group shows no significant difference between the groups, *P* > 0.05.

### 3.4 Effect of DSA on the bone marrow of the anemic mice

**Figure 4:**
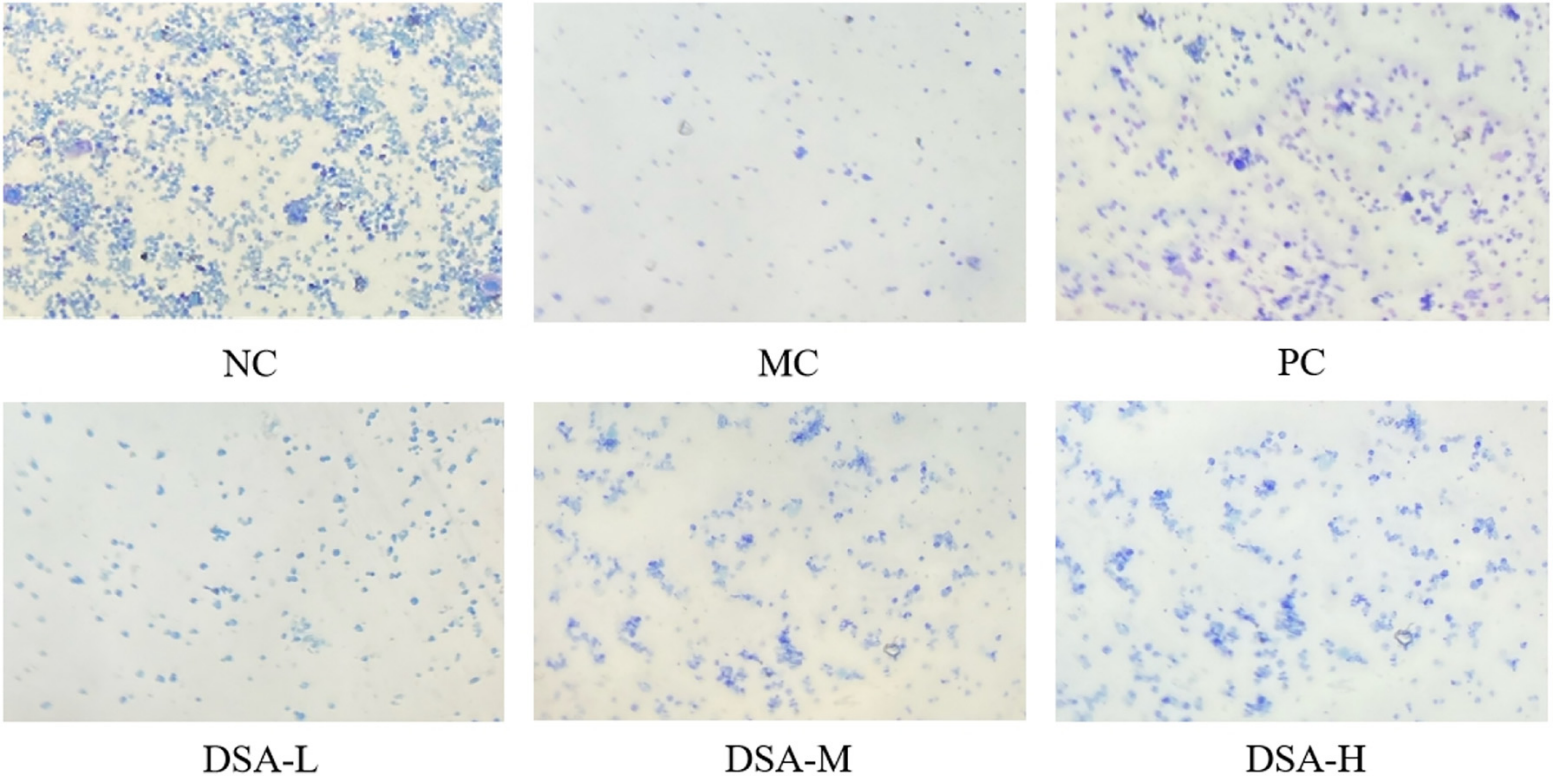
Effect of DSA on the bone marrow images of the anemic mice. Each value represents the mean ± SD (*n* = 10) versus normal control group: ^#^ *^#^P* < 0.01; **versus model control group: **P < 0.01, *P < 0.05.

Bone marrow is a key hematopoetic organ that has different hematopoiesis at different stages of maturity. CP damages hematopoietic microenvironment, resulting in the defect in bone marrow recovery [21]. Figure 4 presents the results. Normal control group bone marrow sampling had normal activities and grew more density on nucleus. Model control group showed a more significant reduction in bone marrow nuclear cells, increased bone marrow steatosis, higher number of non-hematopoietic cells, and an increased bone marrow injury score compared to the normal control group (*P* < 0.01). Each administration group showed active bone marrow tissue proliferation, increased bone marrow cell count, increased hematopoietic tissue, decreased proportional adipose tissue, and increased nuclear cell number were observed relative to the model control group [22]. In mid-high dose group of DSA was found in the mice that showed significant reduction in bone marrow injury score compared to the control group (*P* < 0.05, *P* < 0.01) which shows that DSA decreased bone marrow damage in anemic mice.

### 3.5 Effect of DSA on the bone marrow pathology in the anemic mice

**Figure 5:**
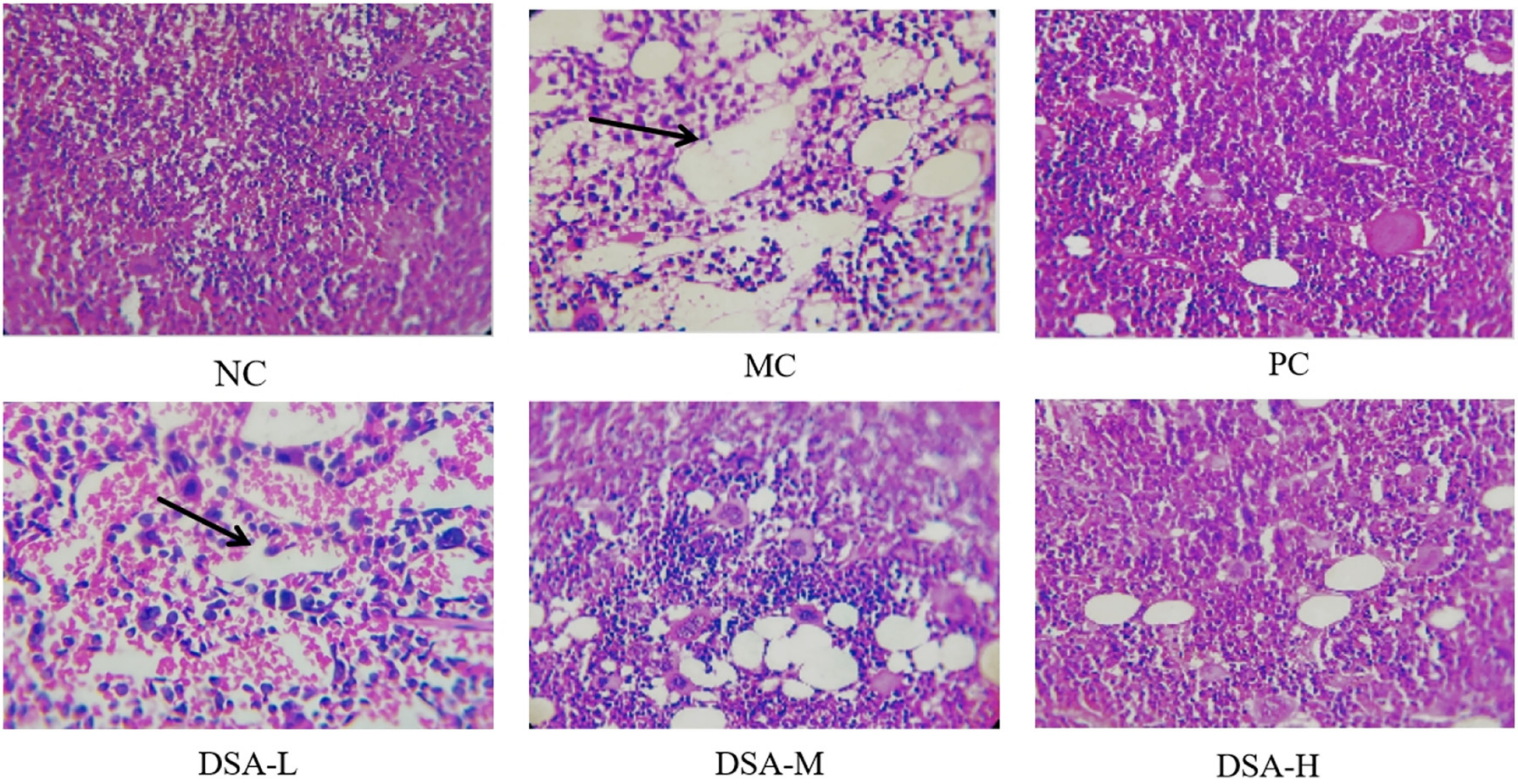
Effect of DSA on bone marrow pathology in the anemic mice (×100). Each value is expressed as the mean ± SD (*n* = 10). ^#^ *^#^P* < 0.01; **P < 0.01, *P < 0.05.

Figure 5 presents the results. In the control group models bone marrow cavities exhibited marked decline in nucleated cells along with an increase in damage levels, blood content rise, fat content increase, decrease in hematopoietic tissue, resulting in a pathological damage score considerably greater than that of normal controls (*P* < 0.01). Mature cells showed resolved maturation disorders, and there was more bone marrow hyperplasia in the positive control group and medium to high dose DSA groups than in the model control group. Granulocyte and erythrocyte morphology was normal with increase in nucleated cells and decrease in lipid drop vacuoles. Furthermore, compared with high-dose DSA group has obvious improvement (*P* < 0.05) and (*P* < 0.01) in score of pathological injury in bone marrow. We can see from our experiment that DSA is capable of inhibiting the growth of the bone fat cell. We acknowledge it will possibly lower certain injury in the bone marrow of mice with anemia.

### 3.6 Effect of DSA on the spleen pathology in the anemic mice

**Figure 6:**
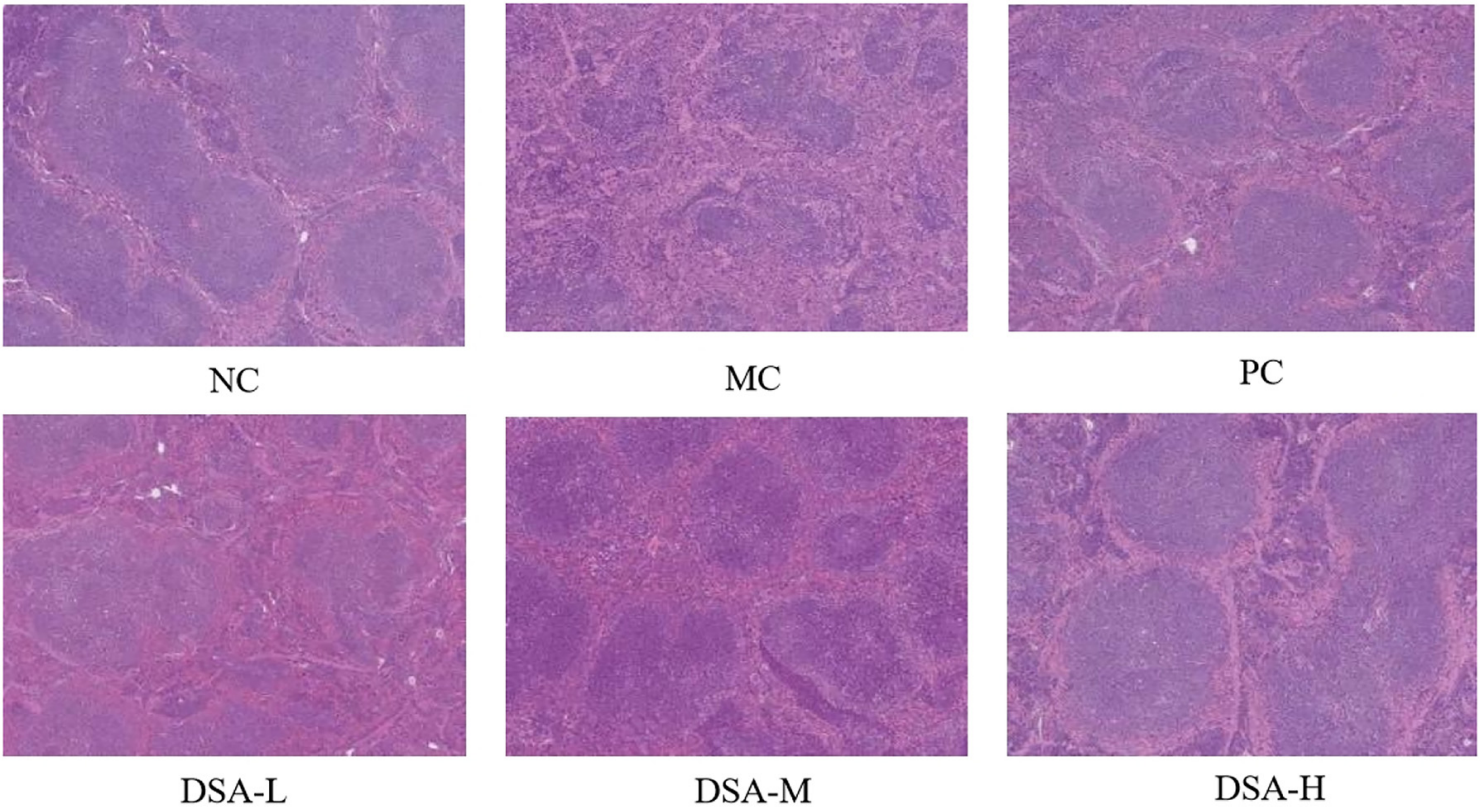
Effect of DSA on spleen pathology in the anemic mice (×400). Each value represents the mean ± SD (*n* = 10). Compared with the normal control group: ^##^ *P* < 0.01; compared with the model control group: **P < 0.01, *P < 0.05.

Spleen is an excellent instance of primary immune hematopoiesis organ. There is no production of blood so bone marrow cells stay in spleen making it large [23]. Figure 6 displays the results. Normal control group: The structure of the spleen can be divided into 2 different parts: the red pulp, and the white pulp. Red pulp: RBCs a lots, splenic corpuscle have. Model control group: The boundary between the red and white pulp within the spleen is less distinct than that in normal control group, the number of lymphocytes has been reduced and there are less splenic corpuscles. There is congestion in the splenic sinus. Positive control group had obviously improvement in the line segment between red pulp and white pulp of the spleen; lymphocyte has risen; the splenic corpuscles are more. The DSA is classified as low, medium, and high and there is a large amount of lymphocyte. White and red pulp are separated. The DSA component shows that spleen damage can be reversed in mice.

### 3.7 Effects of DSA on EPO, TPO and VEGF levels in the serum and bone marrow supernatants of the anemic mice

**Figure 7:**
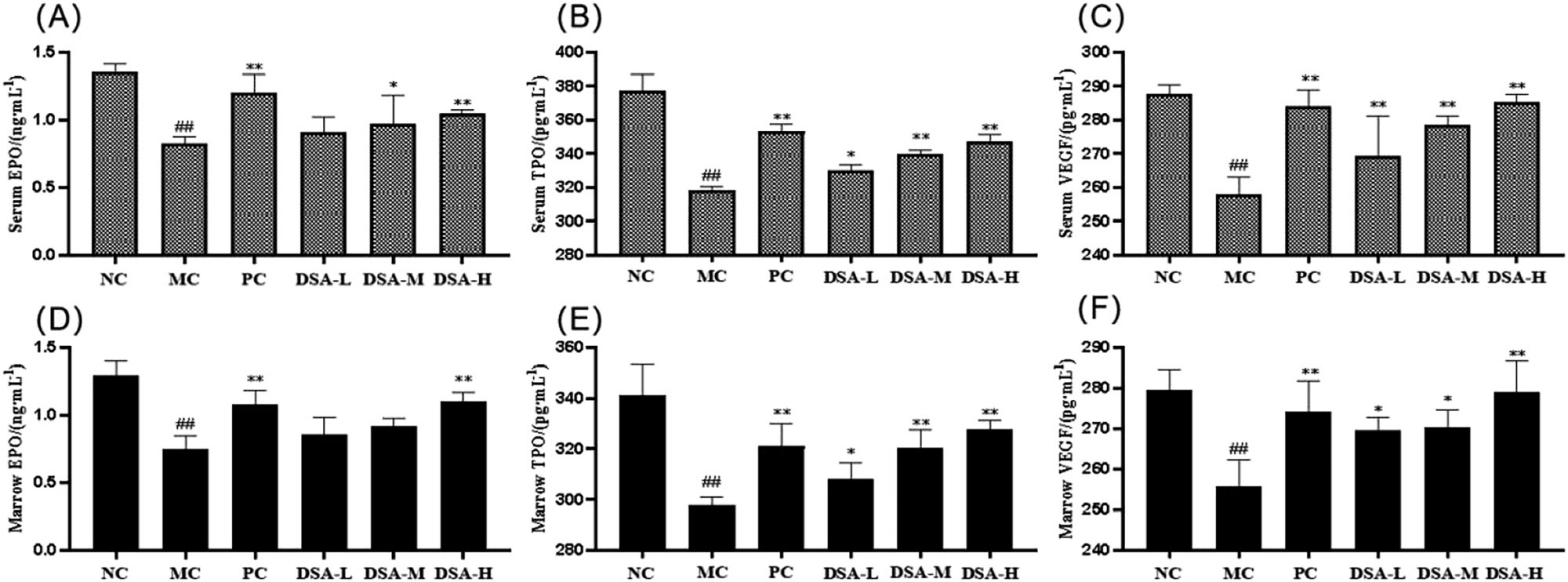
Effects of DSA on the levels of EPO, TPO, and VEGF in the serum and bone marrow supernatants of the anemic mice. Each value represents the mean ± SD (*n* = 10). Compared with the normal control group: *^##^P* < 0.01; compared with the model control group: **P < 0.01, *P < 0.05.

Erythropoietin (EPO) plays a key role in stimulating the growth, differentiation, and maturation of erythroid progenitor cells within the bone marrow. EPO enhances bone marrow hematopoiesis, raises RBC counts, and influences the proliferation, differentiation, and maturation of erythroid progenitor cells [24], 25]. TPO, a key hematopoietic growth factor, is essential for regulating hematopoiesis. TPO can enhance megakaryocyte proliferation and differentiation, facilitate platelet maturation and release, and subsequently aid in the recovery of peripheral blood parameters [26]. Vascular endothelial growth factor (VEGF) stands out as the most potent angiogenic factor, playing a crucial role in driving the formation of new blood vessels [27]. Figure 7 shows the results: preventing endothelial apoptosis, regulating angiogenesis and hematopoiesis, and significantly impacting the body’s hematopoietic function. The levels of EPO, TPO, and VEGF in the serum and bone marrow supernatant of the model control group were markedly lower than those in the normal control group (*P* < 0.01). The positive control group and low-, medium-, and high-dose DSA groups exhibited significantly higher levels of TPO and VEGF in both the serum and bone marrow supernatant of anemic mice compared to the model-control group (*P* < 0.01; *P* < 0.05). Additionally, the positive control and high-dose DSA groups showed markedly elevated EPO concentrations in both serum and bone marrow supernatant (*P* < 0.01, *P* < 0.05), while the medium-dose DSA group demonstrated a significant increase in serum EPO levels relative to the control group (*P* < 0.05).

## 4 Discussion and conclusoin

Current CP-induced anemia mice mainly include CP injection, low iron diet, ferrous protein diet. CP is used to treat cancer, but this medicine causes anemia as its side effects [28]. CP also has hematologic toxicity, such as inhibiting the proliferation of hematopoietic cells, resulting in decreased erythropoiesis and anemia [6]. On the other hand, the CP induced anemia model is easier to establish and has better stability and success rate compared with normal anemia [29]. The quantitative analysis of anemia severity in a model can also be done through analysis of the mice hemoglobin levels, number of RBC’s and hematocrit.

It is noteworthy that the spleen and thymus are immune organs, and CP can also induce immunodeficiency in mice. Previous studies have demonstrated that ACC can regulate organ indices and effectively ameliorate immunodeficiency through upre-gulating TNF-α and GM-CSF expression in serum, as well as TNF-α and GM-CSF mRNA expression in the spleen [29]. These findings are consistent with the results of our study.

Studies on ACC for the treatment of anemia have been reported with varying methods and objectives. For example, peptides with hematopoietic activity have been isolated from ACC proteins, ACC peptides have been complexed with iron to specifically address iron-deficiency anemia, and metabolomics has been employed to investigate hematopoietic mechanisms. Recent years have seen investigations into natural product-derived anti-anemic agents such as *Salvia miltiorrhiza* and oyster. Network pharmacology approaches have been employed to identify relevant targets and explore the mechanism of action of *S. miltiorrhiza* in anemia treatment [30]. Additionally, the complex formed between oyster protein hydrolysates and iron has been studied to address iron deficiency symptoms, including iron-deficiency anemia [31]. Our study found that DSA exerts a stimulating effect on EPO, TPO, and VEGF in CP-induced anemic mice, modulates thymus and spleen indices, and consequently influences the histopathological characteristics of bone marrow and spleen in anemic mice. DSA shows potential as a therapeutic candidate for ameliorating chemotherapy-induced anemia. We’ll further investigate the reasons behind DSA’s influence, alongside other indicators concerning bone marrow hematopoiesis.
